# Hemoglobin Variants as Targets for Stabilizing Drugs

**DOI:** 10.3390/molecules30020385

**Published:** 2025-01-17

**Authors:** Miroslava Žoldáková, Michal Novotný, Krishna P. Khakurel, Gabriel Žoldák

**Affiliations:** 1 Faculty of Science, Pavol Jozef Šafárik University in Košice, Park Angelinum 19, 040 01 Košice, Slovakia; 2 AURORA R&D s.r.o., Mojmírova 12, 040 01 Košice, Slovakia; 3 Extreme Light Infrastructure ERIC, Za Radnici 835, 25241 Dolni Brezany, Czech Republic

**Keywords:** hemoglobin (Hb), oxygen affinity, allosteric regulation, Bohr effect, 2,3-bisphosphoglycerate (2,3-BPG), genetic variants, thalassemia, sickle cell disease (SCD), oxygen-binding properties, protein engineering

## Abstract

Hemoglobin is an oxygen-transport protein in red blood cells that interacts with multiple ligands, e.g., oxygen, carbon dioxide, carbon monoxide, and nitric oxide. Genetic variations in hemoglobin chains, such as those underlying sickle cell disease and thalassemias, present substantial clinical challenges. Here, we review the progress in research, including the use of allosteric modulators, pharmacological chaperones, and antioxidant treatments, which has begun to improve hemoglobin stability and oxygen affinity. According to UniProt (as of 7 August 2024), 819 variants of the α-hemoglobin subunit and 771 variants of the β-hemoglobin subunit have been documented, with over 116 classified as unstable. These data demonstrate the urgent need to develop variant-specific stabilizing options. Beyond small-molecule drugs/binders, novel protein-based strategies—such as engineered hemoglobin-binding proteins (including falcilysin, llama-derived nanobodies, and α-hemoglobin-stabilizing proteins)—offer promising new options. As our understanding of hemoglobin’s structural and functional diversity grows, so does the potential for genotype-driven approaches. Continued research into hemoglobin stabilization and ligand-binding modification may yield more precise, effective treatments and pave the way toward effective strategies for hemoglobinopathies.

## 1. Introduction

Hemoglobin (Hb) is a crucial protein for oxygen transport in mammals, with a complex structure and function. The cooperative binding of oxygen to Hb involves an equilibrium between two quaternary structures with different oxygen affinities [1,2]. This process is influenced by various factors, including pH (the Bohr effect), organic phosphates, and other allosteric effectors [3,4]. The two-state model proposed by Monod, Wyman, and Changeux has been widely used to explain Hb cooperativity, although some discrepancies have been observed [2].

**Figure 1:**
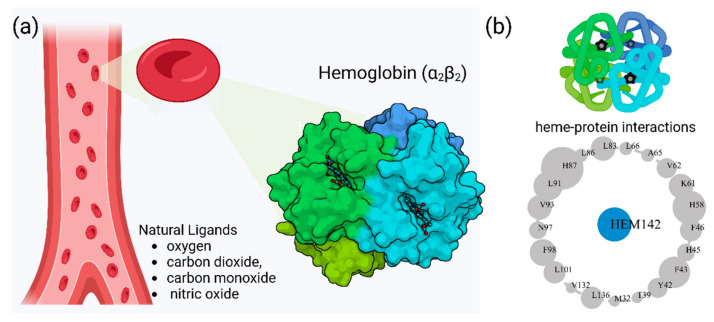
(**a**) Structure of hemoglobin (α2β2): α-subunits are represented in light and dark green, while β-subunits are shown in blue and turquoise. Hemoglobin can interact with natural ligands such as oxygen, carbon dioxide, carbon monoxide, and nitric oxide. (**b**) Heme–protein interactions in hemoglobin, illustrating the key residues in the first layer (L83, L86, L66, A65, V62, K61, H58, F46, H45, F43, Y42, T39, L136, M32, V132, L101, F98, N97, L91, H87, V93) involved in stabilizing the heme group within the protein. Asteroid plot was calculated using web server Protein Contacts Atlas (https://pca.mbgroup.bio/index.html, accessed on 12 August 2024) [21]. Created with www.BioRender.com.

Hb exhibits complex allosteric regulation, crucial for its oxygen-binding function. The interplay between tense and relaxed states governs Hb’s behavior [5,6]. Allosteric effectors, both endogenous and synthetic, modulate Hb’s oxygen affinity by binding to specific sites, altering its structure and function [7,8]. This allosteric control involves both homotropic and heterotropic effects, where ligand binding at one site influences binding at other sites [9]. Protein engineering strategies have explored mutations to optimize Hb’s functional properties, revealing additive effects of interfacial and distal-heme pocket mutations on structure and function [10]. The evolution of Hb has resulted in diverse variants with altered oxygen-binding properties, while maintaining fundamental structural features [11]. Understanding these mechanisms is crucial for developing Hb-based oxygen carriers and treating blood-related disorders [12]. Hemoglobin oxygen affinity is influenced by several physiological ligands (Figure 1): (1) pH aka Bohr effect—a decrease in pH reduces hemoglobin’s oxygen affinity, promoting oxygen release in tissues [3]; (2) CO_2_ levels—increased CO_2_ levels form carbaminohemoglobin, stabilizing the T state and reducing oxygen affinity [13]; (3) NO, which can bind to Hb’s heme groups, forming nitrosylhemoglobin, or to cysteine residues, creating S-nitrosohemoglobin (SNO-Hb) [14,15] and (4) 2,3-Bisphosphoglycerate (2,3-BPG)—this metabolite binds to deoxygenated hemoglobin, stabilizing the T state and promoting oxygen release in tissues. 2,3-Bisphosphoglycerate (2,3-BPG) is a crucial metabolite that binds to hemoglobin, regulating oxygen affinity and delivery to tissues. It binds more strongly to deoxyhemoglobin than oxyhemoglobin, with one binding site per tetramer [16,17,18]. 2,3-BPG levels increase during hypoxia, enhancing oxygen delivery [19]. Its concentration is regulated by complex interactions between glycolysis, the 2,3-BPG shunt, and the pentose phosphate pathway [20]. Feedback inhibition of hexokinase and phosphofructokinase by 2,3-BPG, along with product inhibition of 2,3-BPG synthase, control its steady-state concentration. H^+^ and oxygen effectively regulate 2,3-BPG levels, primarily through hexokinase and phosphofructokinase. The 2,3-BPG shunt also plays a role in stabilizing ATP levels.

Hemoglobin (Hb) is an essential protein for oxygen transport in mammals, with a complex allosteric regulation that enables efficient oxygen delivery to tissues. Genetic mutations affecting hemoglobin structure and function lead to a wide range of disorders, including hemoglobinopathies such as sickle cell disease and thalassemias. These conditions represent significant health challenges worldwide, driving the need for targeted and effective therapeutic strategies.

The objective of this review is to synthesize current knowledge on the physiological roles of hemoglobin and the clinical implications of its variants. We focus on exploring the mechanisms of hemoglobin stability and instability, the impact of genetic diversity, and the therapeutic potential of small molecules and protein engineering. By addressing these aspects, the review aims to provide insights into the development of personalized therapies for hemoglobinopathies. This work also emphasizes the importance of understanding structural dynamics and variant-specific responses.

## 2. Hemoglobin Function and Clinical Variants

The α and β globin genes are located on different chromosomes, with α globin genes on chromosome 16 and β globin genes on chromosome 11. The arrangement and expression of these genes are tightly regulated to ensure proper hemoglobin formation and function. Two main natural variants exist: fetal hemoglobin (HbF, α2γ2) and adult hemoglobin (HbA, α2β2) [22]. HbF has higher oxygen affinity than HbA, facilitating oxygen transfer from mother to fetus [23]. During development, HbF levels decrease from 70% at birth to <1% in adults, while HbA becomes predominant [24,25]. HbA2 (α2δ2) comprises 1.5–3.5% of adult hemoglobin [26]. Genetic factors, including mutations and polymorphisms, can influence HbF production in adulthood [27]. Structural hemoglobinopathies and thalassemias result from mutations affecting globin genes, leading to abnormal or decreased hemoglobin production [28]. These variants can interfere with measurements of glycated hemoglobin, HbA1c, impacting diabetes monitoring [29].

The α- and β-globin gene clusters are characterized by sophisticated genetic regulatory mechanisms designed for expression control during various developmental stages. The regulation process of globin genes was covered in a recent review [30]. The α-globin cluster is located on chromosome 16 at position 16p13.3. The α-globin cluster encompasses key genes including two nearly identical α-globin genes (HBA1 and HBA2) and pseudogenes. These genes are primarily expressed in postnatal and adult life. Additionally, the embryonic ζ-globin genes (HBZ), expressed during the embryonic stage, are also part of this cluster. The entire α-globin cluster spans approximately 30 kilobases and includes regulatory elements crucial for gene expression. The β-globin cluster is situated on chromosome 11 at position 11p15.5, the β-globin cluster extends over about 60 kilobases. It contains several genes essential for hemoglobin production through different stages of development: ε (HBE), expressed in the embryonic stage; two fetal γ-globin genes (HBG1, HBG2); and the adult δ (HBD) and β (HBB) globin genes. The locus control region (LCR), located upstream of these genes, plays a pivotal role in regulating their expression through chromatin looping mechanisms, ensuring that each gene is expressed at the appropriate developmental stage. The regulation of these gene clusters is complex and involves multiple mechanisms. LCRs are key regulatory DNA sequences that interact with globin genes to enhance their transcription. The LCRs achieve this by forming chromatin loops that bring the LCRs into proximity with the genes they regulate, facilitating a high level of gene expression during specific developmental stages. In addition to that several transcription factors and chromatin modifications are involved. Transcription factors, along with various chromatin modifications such as DNA methylation and histone acetylation or methylation, modulate the transcriptional activity of these genes. For instance, during the transition from fetal to adult hemoglobin, specific transcription factors such as BCL11A play a role in repressing γ-globin gene expression while enhancing β-globin gene expression.

Hence, during the embryonic stage, the ε and ζ genes are primarily active, producing embryonic variants of hemoglobin. Next, during the fetal stage, γ-globin genes dominate, producing fetal hemoglobin (HbF), which has a higher affinity for oxygen than adult hemoglobin. During the adult stage, the expression shifts towards the δ and β genes, which contribute to the formation of adult hemoglobin (HbA). The transition from fetal (HbF) to adult hemoglobin (HbA) involves a decrease in the expression of γ-globin coupled with an increase in β-globin production. This switch is critical for adapting the oxygen-carrying capacity of hemoglobin to the lower oxygen environment outside the womb. The regulatory mechanisms behind this switch are crucial for understanding diseases like sickle cell disease and β-thalassemia, where therapeutic strategies often involve reactivating fetal hemoglobin production to counteract the effects of defective β-globin.

In summary, the regulation of α-globin and β-globin gene expression is a complex process involving multiple transcription factors, epigenetic modifications, and chromatin remodeling (see [30] for more details).

### 2.1. Diversity of Genetic Variants of Hemoglobin

Hemoglobin variants are naturally occurring mutations in the hemoglobin molecule that can have significant implications for clinical diagnosis and treatment. According to the World Health Organization, approximately 7% of the global population is affected by hemoglobin variants [31]. Hemoglobin variants can lead to various hematological disorders, such as sickle cell disease and β-thalassemia, which require effective therapeutic strategies. Several approaches have been explored to stabilize hemoglobin variants and mitigate the associated clinical complications. One promising approach is the induction of fetal hemoglobin (HbF) expression. Stabilizing the structure and function of hemoglobin variants is also crucial. The discovery of the alpha-hemoglobin stabilizing protein (AHSP), a chaperone for free alpha-hemoglobin, has provided insights into the importance of balanced globin levels for effective [32]. Targeting the interaction between AHSP and abnormal hemoglobin variants may help stabilize these variants and mitigate the associated pathologies [33]. Another viable option is to explore already-known small-molecule binders of hemoglobin (see Section 2.4). Furthermore, gene therapy approaches, such as CRISPR-Cas9-mediated knockin of the HBB gene, have shown promise in correcting hemoglobin disorders [34]. Lentiviral vector-mediated gene therapy has also demonstrated long-term safety and stability in preclinical models [35,36].

Around 10 years ago, over 1000 naturally occurring hemoglobin variants were identified; these variants show various effects on hemoglobin structure and biochemical properties with varying physiological effects [37]. Since then, several new variants have been found, and their sequences can be retrieved from the UniProt database. UniProt entries for human hemoglobin α and β subunits are 

https://www.uniprot.org/uniprotkb/P69905/entry (accessed on 7 August 2024)

https://www.uniprot.org/uniprotkb/P68871/entry (accessed on 7 August 2024)

In the UniProt database, there are 819 variants of the hemoglobin subunit α and 771 variants of the hemoglobin subunit β (as of 7 August 2024, Figure 2). Assuming homozygotes, there are 631,749 potential combinations of hemoglobin. However, the genetic diversity increases exponentially when considering heterozygotes, resulting in over 10^11^ possible combinations. This number surpasses the current human population on Earth, illustrating the astonishing potential variations in known combinations of hemoglobin variants. The actual diversity is likely much lower because Hb variants are often present in specific geographically constrained sub-populations (see Table 1). For example, HbS is prevalent in Africa and India, thalassemias are common in the Mediterranean, Middle East, and Southeast Asia, HbC is common in West Africa, HbD-Punjab has been found in the Indian subcontinent, HbE is highly prevalent in Southeast Asia, Hb Lepore is prevalent in Southern Italy and Mediterranean populations, Hb Constant Spring (Hb CS) is common in Southeast Asia, Hb Bart’s (γ4) has been found in individuals with α-thalassemia, prevalent in Saudi Arabia and Thailand. Summarizing details can be found in Table 1 and in the main text.

The classification of genetic variants, particularly in hemoglobinopathies, is crucial for accurate diagnosis and clinical management. The American College of Medical Genetics and Genomics (ACMG) guidelines provide a five-category system: pathogenic, likely pathogenic, uncertain significance, likely benign, and benign [38,39]. However, this system has limitations in addressing the spectrum of variant effects. An expanded seven-category system, including “predisposing” and “likely predisposing”, has been proposed to better classify variants in disease-causing genes [38,40]. Variant reclassification, driven by new evidence, can significantly impact patient care, with reclassification rates ranging from 3.6% to 58.8% [41]. For hemoglobinopathies, molecular genetic testing is essential for accurate diagnosis and genetic counseling [42,43]. The clinical implications of variant classification are profound, affecting organ surveillance, prophylactic surgery, and cascade testing [44].

Hemoglobinopathies are a group of disorders affecting the hemoglobin molecule. These disorders typically result from genetic mutations that lead to abnormal structure or production of hemoglobin, causing various health problems ranging from mild anemia to severe complications (Table 1). For example, HbM variants lead to methemoglobinemia, where iron in the heme group is oxidized to the ferric form, impairing oxygen delivery. Methemoglobinemia is a blood disorder characterized by elevated levels of methemoglobin, which impairs oxygen delivery to tissues [45]. It can be inherited or acquired, with the latter being more common and often caused by medications or chemicals [46]. Symptoms include cyanosis unresponsive to oxygen therapy and chocolate-colored blood [47].

Diagnosis is confirmed through co-oximetry, arterial blood gas analysis, and serum methemoglobin levels [48]. Treatment involves removing the offending agent, administering oxygen, and using methylene blue as an antidote in severe cases [49]. Dapsone is a common cause of acquired methemoglobinemia, while benzocaine spray has been associated with severe cases [50]. In pediatric patients, underlying hematologic diseases and G6PD deficiency can predispose to methemoglobinemia [51]. Increased awareness and primary prevention efforts are crucial to reduce morbidity and mortality associated with this condition [50].

**Table 1:** Summary of selected hemoglobin variants, their genetic background, clinical presentations, and diagnostic/therapeutic considerations.

Hb Variant	Mutation	Geo. Distribution	Clinical Features	Diag. Method	Notes
HbC	β-globin gene mutation (Glu → Lys at position 6) [52]	Common in West Africa	Mild chronic hemolysis, splenomegaly, jaundice in homozygotes; compound heterozygosity with HbS can worsen severity [52,53]	Hemoglobin electrophoresis, visualization of HbC crystals [54,55]	Affects RBC rigidity and may influence acquired immunity to malaria [56]
HbD-Punjab	β-globin gene mutation at codon 121 (Glu → Gln) [57]	Relatively low (~0.06% in one study) [57]; found in Indian subcontinent and other regions	Generally asymptomatic in heterozygotes; homozygotes may have mild hemolytic anemia and splenomegaly; severity increases when co-inherited with SCD or thalassemias [31,57,58,59,60,61,62]	Hemoglobin electrophoresis, molecular studies [60]	Treatment may include transfusions, hydroxyurea; HbSD-Punjab can mimic sickle cell disease [59]
HbE	β-globin gene mutation (Glu → Lys at position 26) [63]	Common in Southeast Asia; frequencies up to 0.433 in some Lao populations [64]	Variable severity from asymptomatic to severe anemia when co-inherited with β-thalassemia; altered redox properties and susceptibility to oxidative damage [65,66,67,68]	Hemoglobin electrophoresis, molecular analysis	Origin linked to malarial selection; minimal allosteric changes but altered redox properties [63,65]
Hb Bart’s (γ4)	α-globin gene deletions leading to excess γ chains (γ4 tetramers) [69]	High rates in regions with α-thalassemia (e.g., Saudi Arabia, Thailand) [70,71]	Indicates α-thalassemia severity; elevated levels correspond to number of α-globin gene deletions; high oxygen affinity but no cooperativity [69,72,73,74]	Quantification in cord blood; spectroscopic studies [74,75]	In some cases, elevated Hb Bart’s may be due to developmental asynchrony rather than true α-thalassemia [76]
Hb Chesapeake	α92 Arg → Leu mutation [77,78]	Reported in certain families; not widely prevalent	High oxygen affinity variant causing mild erythrocytosis; reduces tissue oxygen delivery, triggering increased RBC production [77,78,79,80]	Hemoglobin electrophoresis, oxygen dissociation studies, molecular analysis	Similar to other high-affinity variants (e.g., Hb Kempsey); fetal form also has increased O_2_ affinity [77,78]
Hb Lepore	Fusion of δ and β globin genes due to unequal crossover [81,82]	Prevalent in Southern Italy, found globally due to migration [83]	β-thalassemia minor phenotype in carriers; severe anemia in homozygotes or with co-inherited β-thalassemia [83,84,85,86]	Hematological, biochemical, and molecular analyses [82]	Variants include Lepore-Boston, Lepore-Hollandia, and Lepore-Washington-Boston; new variant Hb Lepore-Hong Kong identified [87]
Hb Constant Spring (CS)	Mutation in α2-globin termination codon, elongating α-chain [88]	Common in Southeast Asia; gene frequencies vary from 0.008 in Thailand to 0.143 in Vietnam [89,90]	Can lead to thalassemia intermedia when combined with α-thalassemia; rare homozygous cases cause fetal anemia and hydrops [91,92]	Selective enzymatic amplification of α2-globin DNA; hemoglobin electrophoresis [93]	Interferes with glycated hemoglobin measurements; important for genetic counseling [94]
Hb O-Arab	β121 Glu → Lys mutation [95,96]	Found in populations from the Middle East, North Africa, African Americans, West Africans [95,97,98]	Generally mild in homozygous form, but compound heterozygosity (e.g., Hb S/O Arab) can cause severe disease [95,99]	Hemoglobin electrophoresis with specialized techniques [95]	Management may involve transfusions and splenectomy [100]
Hb Seal Rock	Extended α-chain variant [101]	Rare, limited reports	Associated with mild Hb H disease and α-thalassemia-2 trait [101]	Hemoglobin electrophoresis, molecular studies	Impact depends on mutation location within the gene [102]
Hb Indianapolis	Rare, unstable β-globin variant [103]	Reported in a Brazilian patient [103]	Moderate hemolytic anemia and renal damage [103]	Routine DNA sequencing of globin genes [104]	Highlights the growing diversity of novel β-chain variants
Unstable Variants (e.g., Hb Madrid, Hb Showa-Yakushiji, Hb Santander, Hb Yokohama, Hb Seattle, Hb Miami, Hb Hershey, Hb Abington)	Various mutations in β-globin affecting amino acid positions [105,106,107,108,109,110,111]	Reported in disparate geographic locations (Spain, Republic of Korea, India, Japan, etc.) [105,106,107,108,109,110]	Mild to moderate hemolytic anemia; severity often increases with co-inherited thalassemia mutations [105,106,107,108,109,110,111]	Hemoglobin electrophoresis, DNA sequencing, RBC morphology analysis	Demonstrate clinical severity range and genetic complexity of unstable hemoglobin variants [104,105,106,107,108,109,110,111]

Sickle cell disease (SCD) is a genetic disorder caused by a single nucleotide mutation in the β-globin gene, resulting in the production of abnormal hemoglobin S (HbS) [112,113]. This E6V mutation leads to HbS polymerization under low oxygen conditions, causing red blood cell (RBC) sickling and hemolysis [112,114]. SCD is characterized by chronic inflammation, oxidative stress, and vascular dysfunction, contributing to various complications such as stroke, pulmonary hypertension, and renal disorders [113,115]. The disease affects millions worldwide, with higher prevalence in Africa and India [114]. Recent advancements in care, including newborn screening, penicillin prophylaxis, and hydroxyurea treatment, have improved patient outcomes [116,117]. Ongoing research explores gene editing techniques like CRISPR/Cas9 to target the β-globin gene in hematopoietic stem cells as a potential therapeutic approach [118]. Gene therapy approaches using βT87Q-globin have shown anti-sickling effects by reducing endogenous βS-globin expression [119]. These advancements in antisickling agents and therapies offer new possibilities for treating sickle cell disease.

Thalassemias are inherited disorders of hemoglobin synthesis, characterized by deficient production of globin chains [120,121]. They are prevalent in the Mediterranean, Middle Eastern, and Southeast Asian regions [122,123]. Alpha and beta thalassemias are the most common types, resulting from mutations in α and β globin genes, respectively [124]. Symptoms range from asymptomatic in carriers to severe anemia requiring regular blood transfusions in major forms [125]. Complications include iron overload, cardiac issues, and endocrine disorders [126]. Treatment options include blood transfusions, iron chelation therapy, and bone marrow transplantation [122]. Gene therapy and editing strategies are emerging as potential curative treatments [120]. Prevention through premarital screening and prenatal diagnosis is crucial in reducing the prevalence of thalassemia [126]. Despite its significant impact, thalassemia is often underrecognized in global health assessments [121].

Hemoglobin C (HbC) is a common structural variant of normal hemoglobin, resulting from a mutation in the beta-globin gene [52]. HbC is less soluble than normal hemoglobin, leading to increased rigidity of red blood cells [53]. This can cause mild chronic hemolysis, splenomegaly, and jaundice in homozygous individuals [52]. HbC interacts more strongly with erythrocyte membranes than normal hemoglobin [127]. While HbC disease is generally mild, its inheritance with other hemoglobinopathies like HbS can have serious consequences [52]. Both HbC and HbS may affect the development of acquired immunity against malaria [56]. Efforts have been made to develop simple, inexpensive methods for detecting HbC in resource-limited settings, including microscopic visualization of HbC crystals [54,55]. These methods could potentially determine zygosity and aid in diagnosis in underdeveloped countries where HbC is prevalent.

Hemoglobin D-Punjab (HbD-Punjab) is a variant resulting from a mutation in codon 121 of the β-globin gene [57]. While heterozygous HbD-Punjab is generally asymptomatic, homozygous cases can cause mild hemolytic anemia and splenomegaly [57,128]. The clinical presentation of HbD-Punjab can vary, especially when coinherited with other hemoglobinopathies or thalassemias [58,60]. HbSD-Punjab, a compound heterozygous condition, can lead to severe symptoms resembling sickle cell disease [59]. HbD-Punjab/β-thalassemia combinations may result in moderate to severe anemia [61,62,128]. Diagnosis typically involves hemoglobin electrophoresis and molecular studies [60]. Treatment options include blood transfusions and hydroxyurea, which have shown promise in managing symptoms [59]. The prevalence of HbD-Punjab is relatively low, estimated at 0.06% in one study [57].

Hemoglobin E (HbE), a common β-globin variant in Southeast Asia, results from a Glu26Lys mutation [63,64]. This variant exhibits extended linkage disequilibrium and likely arose 1240–4440 years ago due to malarial selection [63]. HbE frequencies reach up to 0.433 in some Lao populations [64]. While HbE shows minimal allosteric changes, it demonstrates altered redox properties, including decreased nitrite reductase activity and accelerated reduction by cysteine [65]. HbE is more susceptible to oxidative damage, especially in the presence of free α subunits [66]. When co-inherited with β-thalassemia, HbE can cause severe clinical manifestations [67]. However, its association with protection against cerebral malaria remains inconclusive [68]. HbE disorders present a wide range of clinical severity, from asymptomatic carriers to severe anemia [129].

Hemoglobin [130] Bart’s (γ4) is a homotetrameric hemoglobin found in individuals with α-thalassemia [69]. It lacks cooperativity and has a higher oxygen affinity than adult or fetal hemoglobin. Crystal structures reveal that Hb Bart’s resembles the R state of adult hemoglobin [69,72]. Quantification of Hb Bart’s in cord blood accurately indicates α-thalassemia status, with levels correlating to the number of deleted or inactivated α-globin genes [73,74]. The prevalence of Hb Bart’s varies among populations, with high rates reported in Saudi Arabia and Thailand [70,71]. Spectroscopic studies show that Hb Bart’s is structurally similar to Hb H (β4) [75]. In some populations, elevated Hb Bart’s may result from developmental asynchrony rather than α-thalassemia [76].

Hemoglobin Chesapeake (α92 Arg → Leu) is a high-oxygen-affinity hemoglobin variant associated with erythrocytosis [77,78]. This mutation occurs at the α1β2 interface, similar to other high-affinity variants like Hb Wood and Hb Malmö [131]. The increased oxygen affinity results from an early conversion from the T to R state during ligand binding [77]. Hb Chesapeake causes mild erythrocytosis in carriers due to reduced oxygen delivery to tissues, necessitating increased red cell production [79]. This mechanism is also observed in other high-affinity variants like Hb Kempsey [132]. The fetal form of Hb Chesapeake (α2Chesγ2) also exhibits increased oxygen affinity, suggesting similar conformational changes in the γ chain [78]. Generally, high-affinity hemoglobin variants impair the formation of a stable T state or modify the heme environment, leading to compensatory erythrocytosis [80].

Hemoglobin Lepore is a structural variant formed by the fusion of δ and β globin genes, resulting from unequal crossover events [81,82]. It is associated with a β-thalassemia minor phenotype in carriers and can lead to severe anemia in homozygotes or compound heterozygotes with β-thalassemia [83,84]. Several variants exist, including Lepore-Boston, Lepore-Hollandia, and Lepore-Washington-Boston [85,86]. The condition is prevalent in Southern Italy and has spread globally due to migration [83]. Diagnosis requires hematological, biochemical, and molecular analyses [82]. Hb Lepore can interact with other hemoglobinopathies, resulting in various clinical phenotypes [86]. Recently, a novel variant, Hb Lepore-Hong Kong, was identified in a Chinese family [87]. Accurate diagnosis is crucial for genetic counseling and prenatal screening in affected populations.

Hemoglobin Constant Spring (Hb CS) is a variant caused by a mutation in the α2-globin gene termination codon, resulting in an elongated α-chain [88]. It is prevalent in Southeast Asian populations, with gene frequencies ranging from 0.008 in Thailand to 0.143 in Vietnam [89,90]. Hb CS can lead to thalassemia intermedia when combined with α-thalassemia [91]. Diagnosis is challenging due to low amounts of the mutant hemoglobin, but selective enzymatic amplification of α2-globin DNA allows for accurate detection [93]. Hb CS can interfere with glycated hemoglobin measurements using boronate affinity chromatography [94]. In rare cases, homozygous Hb CS can cause fetal anemia and hydrops [92]. Understanding Hb CS is crucial for genetic counseling and prenatal diagnosis in affected populations. Hb O-Arab (Glu121Lys) causes mild hemolytic anemia in populations from the Middle East and North Africa.

Hemoglobin O Arab is a rare abnormal hemoglobin variant characterized by a β121Glu → Lys mutation [95,96]. It can occur in homozygous form or in combination with other hemoglobinopathies. The homozygous form is generally well-tolerated [97,100], while compound heterozygous forms, particularly Hb S/O Arab, can result in severe clinical manifestations like sickle cell anemia [95,99]. Hb S/O Arab patients may experience acute chest syndrome, vasoocclusive events, and other complications [95]. Diagnosis requires specific electrophoresis techniques due to Hb O Arab’s migration patterns [95]. The condition has been reported in various populations, including African Americans, West Africans, and Arabs [95,97,98]. Treatment may involve transfusions and splenectomy in cases of hypersplenism [100].

Hb Seal Rock, an extended α-chain variant, is associated with mild Hb H disease and α-thalassemia-2 trait [101]. Some α-chain variants can lead to chronic hemolytic anemia, with the mutation’s location within the gene sequence playing a crucial role in determining its effect [102]. Hb Indianapolis, a rare and slightly unstable β-globin variant, was reported to cause moderate hemolytic anemia and renal damage in a Brazilian patient [103]. Routine DNA sequencing of α- and β-globin genes has revealed numerous novel mutations, including 11 new β-chain variants, 15 α-chain variants, 19 β-thalassemia mutations, and 15 α+-thalassemia mutations, highlighting the genetic diversity in hemoglobinopathies [104].

Several unstable hemoglobin variants have been identified that cause mild to moderate hemolytic anemia. Hb Madrid (β115Ala → Pro) was found in a Spanish boy and a Korean family, resulting in moderately severe hemolytic anemia [105,106]. Hb Showa-Yakushiji (β110Leu → Pro) was observed in four unrelated Indian patients [107]. Other variants include Hb Santander (β34Val → Asp) in a Spanish patient [108], Hb Yokohama (β31Leu → Pro) in a Japanese family [109], and Hb Seattle (β76Ala → Glu) in a mother and her two sons [110]. Hb Miami (β116His → Pro) and Hb Hershey (β70Ala → Gly) were found in association with beta-thalassemia mutations, while Hb Abington (β70Ala → Pro) was another unstable variant [111]. These variants demonstrate the range of clinical severity observed with unstable hemoglobin mutations, particularly when combined with thalassemic mutations.

### 2.2. Hemoglobin Instability and Heinz Bodies

Heinz bodies are aggregates of denatured hemoglobin that form within red blood cells (RBCs) as a result of oxidative stress or intrinsic instability of hemoglobin. Their presence is indicative of various hemolytic conditions and can be associated with unstable hemoglobin variants, such as those seen in hemoglobinopathies. The formation of Heinz bodies is a complex process influenced by multiple factors, including oxidative damage, genetic mutations, and environmental toxins. This synthesis aims to explore the stability of hemoglobin in relation to Heinz body formation, drawing on a range of studies that elucidate the mechanisms and implications of this phenomenon.

The historical context of Heinz body formation dates back to the late 19th century when Robert Heinz first described these inclusions in cells subjected to oxidative stress. Since then, research has established that Heinz bodies are not exclusive to unstable hemoglobinopathies; they can also arise in conditions such as glucose-6-phosphate dehydrogenase (G6PD) deficiency, where oxidative stress leads to hemolytic anemia [133]. In cases of unstable hemoglobin variants, such as those documented in hemoglobin Montreal II, the instability of the hemoglobin molecule can precipitate under physiological conditions, resulting in Heinz body formation. This instability is often exacerbated by factors such as viral infections, which can further complicate the clinical picture by increasing hemolysis [133].

The biochemical basis for Heinz body formation involves the denaturation of hemoglobin, which can occur due to oxidative damage from various sources, including environmental toxins. For instance, exposure to polycyclic aromatic hydrocarbons (PAHs) has been shown to induce oxidative injury to hemoglobin, leading to the aggregation of denatured hemoglobin into Heinz bodies [134]. This oxidative damage is not limited to specific species; studies have documented similar phenomena in avian species, where exposure to oil pollutants resulted in the formation of Heinz bodies and subsequent hemolytic anemia [135]. The presence of Heinz bodies in these contexts serves as a biomarker for oxidative stress and cellular damage.

In addition to environmental factors, genetic mutations play a crucial role in the stability of hemoglobin. Variants such as Hb Volga exhibit decreased stability, leading to chronic hemolytic anemia characterized by the formation of Heinz bodies composed of precipitated hemichromes and heme-free globins [136]. More than 100 hemoglobin variants have been identified with reduced stability, highlighting the genetic underpinnings of this condition [136]. Furthermore, the role of molecular chaperones, such as the alpha hemoglobin stabilizing protein (AHSP), has been implicated in maintaining hemoglobin stability. AHSP assists in the proper folding of α-globin chains and prevents their aggregation, thereby reducing the likelihood of Heinz body formation [137].

The physiological implications of Heinz body formation are significant, as these inclusions can compromise the functionality of RBCs. The presence of Heinz bodies can lead to increased erythrocyte fragility and a shortened lifespan, as cells containing these aggregates are often removed from circulation by the spleen [138]. This process can result in hemolytic anemia, characterized by fatigue and decreased oxygen-carrying capacity [139]. The clinical manifestations of Heinz body-related hemolytic anemia can vary, with some individuals exhibiting mild symptoms while others may experience severe anemia requiring medical intervention.

Diagnostic approaches to identify Heinz bodies typically involve supravital staining techniques, which can reveal these inclusions in RBCs. For instance, the use of brilliant cresyl blue staining has been shown to enhance the visibility of Heinz bodies, particularly in cases where oxidative damage has occurred [140]. This staining method can be particularly useful in neonatal populations, where Heinz bodies may be present but not readily detected without appropriate staining protocols [140]. The identification of Heinz bodies in clinical samples can provide valuable insights into the underlying etiology of hemolytic anemia and guide further diagnostic testing, such as hemoglobin electrophoresis or high-performance liquid chromatography (HPLC) to characterize abnormal hemoglobin variants [133].

The formation of Heinz bodies is also influenced by dietary factors and exposure to certain plants known to induce oxidative stress. For example, garlic extracts have been shown to deplete thiol levels in erythrocytes, leading to increased susceptibility to oxidative damage and subsequent Heinz body formation [141]. Similarly, the ingestion of Allium species, such as onions, has been documented to cause oxidative damage to hemoglobin, resulting in the precipitation of denatured hemoglobin and the formation of Heinz bodies in affected animals [142]. These findings underscore the importance of environmental and dietary factors in the pathogenesis of hemolytic anemia associated with Heinz body formation.

In summary, the stability of hemoglobin is a critical factor in the formation of Heinz bodies, with both genetic and environmental influences playing significant roles. Unstable hemoglobin variants, oxidative stress from environmental toxins, and dietary factors can all contribute to the denaturation of hemoglobin and the subsequent aggregation into Heinz bodies. The presence of these inclusions serves as a marker for hemolytic anemia and highlights the need for comprehensive diagnostic approaches to identify underlying causes and guide appropriate management strategies. Future research should continue to explore the molecular mechanisms underlying hemoglobin stability and the factors that contribute to Heinz body formation in various populations.

To validate destabilizing mutations in hemoglobin, further genetic testing is needed. Additionally, it can provide some information for treatment choices by recommending the possible use of specific stabilizers or antioxidants to shield red blood cells, lessen oxidative damage, and enhance patient outcomes [143,144,145]. Of course, hemoglobin genetic variants can differ in their susceptibility to oxidation. Certain unstable variants are more likely to undergo oxidation or lose the heme. Losing the heme group, the heme-free globin chains can eventually result in the production of Heinz bodies. Since they exhibit a variety of inclinations to form these inclusions under oxidative stress, approximately 3.8% of α-globin and 11% of β-globin variations are regarded as unstable [143,146]. This link emphasizes how Heinz bodies reflect the distinct molecular features of particular hemoglobin types. In this review, we highlight Heinz bodies in connection with hemoglobin structural and genetic variations. It might be the case that Heinz bodies themselves might not be the main targets of treatment.

However, Heinz bodies highlight their role as clinical indicators. Their function serves to emphasize how crucial it is to take structural weaknesses and oxidative stress into account when creating management or treatment plans for hemoglobin-related illnesses. In general, hemoglobin structural instability can lead to denaturation and precipitation, resulting in the formation of Heinz bodies, which are inclusions of denatured hemoglobin in red blood cells. This instability is often due to amino acid substitutions or deletions that disrupt the hemoglobin structure, enhancing oxidation to methemoglobin and subsequent conversion to hemichrome [143]. The loss of heme, particularly from beta chains, is a critical factor in Heinz body formation [146,147]. The presence of Heinz bodies can cause hemolytic anemia due to mechanical obstruction in microcirculation and oxidative damage to red cell membranes [130,148]. Additionally, factors such as temperature and oxidative stress can exacerbate the formation of Heinz bodies [144]. Oxidative stress occurs when there is an imbalance between reactive oxygen species (ROS) and the body’s antioxidant defenses. Hemoglobin is particularly vulnerable to oxidative damage, which can lead to hemolysis. This oxidative damage is exacerbated in conditions such as thalassemia and sickle cell disease. Mechanisms leading to Heinz body formation include oxidative damage, genetic mutations, chemical exposure, and enzyme deficiencies. Reactive oxygen species (ROS) can oxidize hemoglobin, causing it to denature and form Heinz bodies. This oxidative stress is common in conditions with an elevated level of ROS, such as inflammation or chronic diseases.

In summary, Heinz bodies are microscopic clusters that form when hemoglobin oxidizes and becomes unstable. They frequently serve as a precursor to hemolytic disorders. Their presence in red blood cells may indicate certain underlying problems, such as hemolysis caused by genetic hemoglobin variations that induce instability.

As mentioned above, genetic factors significantly influence the stability of hemoglobin. Mutations in the genes encoding hemoglobin can lead to structurally unstable hemoglobin variants, making them more susceptible to denaturation and resulting in disorders such as thalassemias and hemoglobinopathies. These genetic mutations can affect the function and stability of hemoglobin molecules, leading to various clinical manifestations. Out of the 819 known variants of the hemoglobin subunit α, 31 are classified as unstable. This represents approximately 3.8% of all α subunit variants. In contrast, 85 out of 771 known variants of the hemoglobin subunit β are considered unstable, accounting for about 11% of the β subunit variants. This comparison highlights a significant difference in the frequency of unstable variants between the two subunits. The frequency of unstable variants in the β subunit is nearly three times higher than that in the α subunit, suggesting that the β-globin gene may be more susceptible to mutations that result in instability or that the structural constraints of the β subunit allow for more potentially unstable configurations.

The human α-globin gene cluster, including HBA1 and HBA2, is located on chromosome 16 at position 16p13.2-pter [149,150]. This region is extremely gene-rich, containing 100 confirmed and 20 predicted genes [151]. The α-globin locus is highly polymorphic, with 15 dimorphic and 2 multiallelic genetic markers identified [152]. Upstream regulatory elements, particularly HS-40, play crucial roles in α-globin gene expression [153]. α-Thalassemia, a disorder of reduced α-chain synthesis, is often caused by large deletions within the gene cluster [154]. Interestingly, a 62 kb deletion upstream of the α-globin genes can also cause α-thalassemia without directly affecting the genes themselves [155]. The α-globin locus serves as an ideal genetic marker for constructing human linkage maps and studying the molecular genetics of the cluster [152].

The β-globin gene cluster, located on chromosome 11 at position 11p15.5, includes the HBB gene, which encodes the β-globin chain of adult hemoglobin [156,157]. This cluster comprises several genes, including HBD, which encodes the δ-globin chain, and undergoes critical expression switches during development [158]. The precise localization of the β-globin gene has been confirmed through various studies, indicating its location within the 11p15.4 to 11p15.5 region [159,160,161]. The regulation of these genes is crucial for understanding hemoglobinopathies, with recent findings highlighting the role of factors like BCL11A in silencing fetal hemoglobin [162]. Overall, the β-globin gene cluster is essential for normal hemoglobin function and is implicated in disorders such as sickle cell anemia and β-thalassemia [157].

The observed difference in the frequency of unstable variants between the α and β subunits, 3.8% vs. 11%, may be due to several factors. One would expect that the α-globin gene cluster location on chromosome 16 and its gene duplication may confer additional stability, resulting in a higher chance of accepting unstable variants compared to the β-globin gene cluster on chromosome 11. The presence of two nearly identical genes (HBA1 and HBA2) in the α-globin cluster means there is a redundancy that can potentially compensate for potential mutations. If one gene is mutated, the other can often continue to produce functional α-globin chains, reducing the impact of any single mutation and enhancing overall stability. This redundancy might act as a genetic buffer, making the α-globin production more resilient to genetic mutations and environmental pressures. However, we found that less unstable variants of α-chain are nearly three-fold less indicating that other factors are responsible for a higher number of unstable β-chain variants. Another factor would be structural differences between the α and β subunits might inherently affect their susceptibility to mutations that lead to instability. While both subunits share a high sequence identity of 43% and 50–60% sequence similarity, they may have distinct tolerance toward mutations constrained by their differences in function.

**Figure 2:**
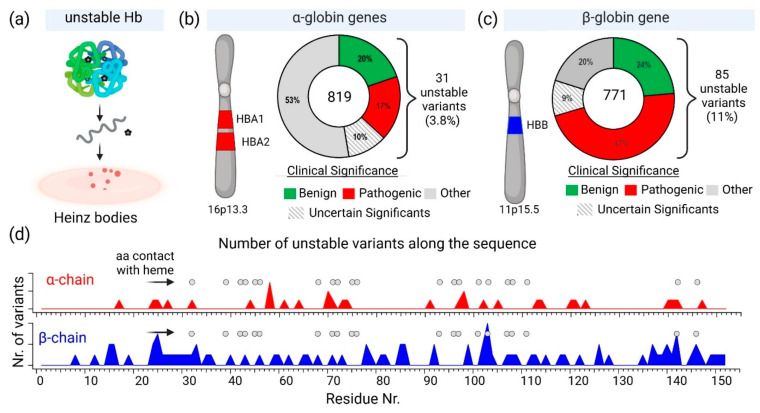
(**a**) Depiction of hemoglobin (Hb) instability leading to the formation of Heinz bodies. Structure of hemoglobin (α2β2): α-subunits are represented in light and dark green, while β-subunits are shown in blue and turquoise. (**b**) Distribution of 819 unstable α-globin variants, with 3.8% classified as unstable, mapped on chromosome 16 (16p13.3) and divided into clinical significance categories: benign, pathogenic, and uncertain significance. (**c**) Distribution of 771 unstable β-globin variants, with 11% classified as unstable, mapped on chromosome 11 (11p15.5), similarly categorized. (**d**) Graph showing the number of unstable variants along the amino acid sequence for both α- and β-globin chains, highlighting regions where residues contact the heme group (grey-filled circles). Created with www.BioRender.com.

The β-globin’s versus α-globin’s specific role and interactions in the hemoglobin tetramer may be the reason for higher counts of unstable mutations. In fact, the β-globin plays a crucial role in hemoglobin structure and function. It forms strong interactions with α-globin to create stable α1β1 dimers, which then assemble into α2β2 tetramers [163]. The β-chains significantly influence oxygen binding characteristics, particularly through alterations in the T state properties [164]. The β112 Cys residue is critical for both homo- (β4) and hetero-tetramer (α2β2) formation, with mutations at this position affecting assembly and stability [165]. The β4 tetramer structure closely resembles the R state of liganded α2β2 hemoglobin, but with some unique interface interactions [166]. The β chains also interact with the cytoplasmic domain of band 3 in erythrocyte membranes, preferentially binding to deoxyhemoglobin [167]. On the other hand, α-globin plays a crucial role in hemoglobin formation and function. The α-Hemoglobin Stabilizing Protein (AHSP) is essential for maintaining α-globin stability and preventing its precipitation [168]. AHSP binds specifically to the G and H helices of α-globin, with the N-terminal part of the H helix being critical for this interaction [169,170]. AHSP acts as a molecular chaperone, facilitating α-globin folding, refolding after denaturation, and incorporation into hemoglobin A [171]. It also protects against oxidative damage and helps maintain the correct ratios of α and β globins during hemoglobin biosynthesis [172]. Mutations in α-globin that impair AHSP binding can lead to protein instability and anemia [173]. The complex interplay between α-globin expression, AHSP, and hemoglobin assembly is crucial for normal erythropoiesis [174]. Hence, we can speculate that these differences between globins may result in observed differences in frequencies of unstable variants that α-globin mutations produce a significant disadvantage, so they are much less tolerated than β-globin unstable variants. In addition, a possible correlation can be found between amino acid residues contacting heme and unstable variants (Figure 2d).

### 2.3. Pharmacological Approaches to Hemoglobin Stabilization and Oxidative Stress in Hemolytic Disorders

Certain molecules and toxins can induce oxidative stress or directly interact with hemoglobin, leading to its denaturation and the formation of so-called Heinz bodies. Even though the below-mentioned small molecules do not directly interact with Hb, they can influence the metabolism of erythrocytes, reduce oxidative stress, or induce other hemoglobin chain expressions, and by doing this, they can provide stability for hemoglobin or prevent sickling.

Glucose-6-phosphate dehydrogenase (G6PD) deficiency, affecting approximately 400 million people worldwide, is a crucial enzyme in protecting red blood cells from oxidative stress [145]. G6PD deficiency can lead to hemolytic anemia, neonatal jaundice, and increased susceptibility to oxidative damage [175]. The deficiency results in reduced NADPH production, compromising the cell’s ability to maintain glutathione in its reduced state [176]. This leads to increased hemoglobin denaturation and ferriheme release, potentially causing hemolysis [177]. Contrary to previous beliefs, NADPH status, rather than glutathione levels, modulates oxidant sensitivity in G6PD-deficient erythrocytes [178]. G6PD deficiency also has broader implications, including increased risk of prenatal and postnatal death, infertility, and teratogenesis [179]. In transfusion medicine, G6PD-deficient blood may pose risks to certain patient populations [180].

Oxidative stress plays a significant role in hemolytic anemias, causing damage to red blood cells and exacerbating symptoms [181]. Antioxidants, such as N-acetylcysteine (NAC) and Vitamin E, have shown promise in reducing oxidative stress and improving hemoglobin levels, particularly in children with transfusion-dependent thalassemia [182]. These antioxidants can inhibit cation pathways responsible for red blood cell dehydration and reduce phosphatidylserine exposure, potentially improving cell rheology and reducing vascular adhesion [183]. Supplementation with vitamin C and NAC has been found to preserve glutathione homeostasis and reduce hemolysis in stored red blood cells [184]. However, high-dose vitamin C and E supplementation may worsen hemolysis in some cases [185]. Combining antioxidants with iron chelators may provide substantial improvements in the pathophysiology of hemolytic anemias, especially thalassemia [186].

Hemin is a pharmacological agent used in treating acute porphyrias, which are metabolic disorders characterized by heme biosynthesis defects. It functions primarily by inhibiting delta-aminolevulinic acid synthase (ALAS-1), the rate-limiting enzyme in heme production, thereby reducing the accumulation of toxic heme precursors like delta-aminolevulinic acid [187,188]. Hemin administration has been shown to alleviate symptoms during acute attacks by restoring heme levels and downregulating ALAS-1 activity [189,190]. Additionally, heme’s feedback inhibition on ALAS-1 is mediated through various mechanisms, including proteolytic degradation of the enzyme [191]. The clinical efficacy of hemin underscores its role in managing acute porphyrias, as it directly addresses the biochemical disturbances underlying these conditions [192,193].

Hydroxyurea is a promising treatment for sickle cell disease, primarily due to its ability to induce fetal hemoglobin (HbF) production [194,195,196]. HbF inhibits the polymerization of sickle hemoglobin (HbS), reducing disease severity [195]. Hydroxyurea works by perturbing erythroid precursor maturation and potentially through nitric oxide-dependent mechanisms [197]. Clinical studies have shown that hydroxyurea treatment increases HbF levels, reduces hemolysis, and decreases the frequency of vaso-occlusive crises and hospitalizations [198,199]. The drug also improves red cell survival and reduces sickling at partial oxygen saturation [200]. While hydroxyurea’s effects can be inconsistent, it has been shown to reduce morbidity and mortality in adults with sickle cell anemia [194]. Ongoing research is exploring its use in children and in combination with other HbF-inducing agents [195,201].

Mitapivat, an oral pyruvate kinase activator, has shown promising results in treating various hemoglobinopathies. In pyruvate kinase deficiency, it increased hemoglobin levels and reduced transfusion burden, with sustained long-term effects [202,203]. For non-transfusion-dependent α- and β-thalassemia, mitapivat improved hemoglobin levels, markers of erythropoiesis, and hemolysis [204,205,206]. In sickle cell disease, it demonstrated improvements in anemia, hemolysis, and hemoglobin S polymerization kinetics [207]. Mitapivat’s mechanism of action involves increasing ATP production in red blood cells, which may help stabilize hemoglobin and improve cell integrity [208]. The drug has shown a favorable safety profile across studies, with common adverse events being generally mild and transient [203,204]. These findings suggest that mitapivat may represent a novel therapeutic approach for various hereditary anemias [209].

### 2.4. Small-Molecule Binders to Hemoglobin

Hemoglobin is also a direct drug target and, hence, several classes of molecules were found to bind and stabilize hemoglobin, which should improve oxygen delivery in patients with hemoglobinopathies such as sickle cell disease and β-thalassemia. Pharmacological chaperones are drugs that stabilize hemoglobin by promoting proper folding and preventing denaturation. These agents help maintain hemoglobin’s functional state under stress conditions, reducing the likelihood of hemolysis (see above). Most of the development of drugs targeting hemoglobin focus on sickle cell diseases, which is caused by a single mutation that results in the substitution of valine for glutamic acid in the sixth position of the β-globin chain of hemoglobin, leading to the polymerization of hemoglobin S upon deoxygenation. Peptides and amino acids stabilizing hemoglobin have been summarized in detail in a recent review [210]. Recent advances in SCD treatment have expanded beyond the traditional therapies of hydroxyurea and L-glutamine. New approaches target various aspects of SCD pathophysiology, including HbS polymerization, cellular adhesion, inflammation, and oxidative stress [211]. The FDA has approved three new drugs: crizanlizumab, voxelotor, and L-glutamine, which have shown efficacy in reducing pain crises and improving hemoglobin levels [212]. Emerging therapies include peptide-based inhibitors of HbS polymerization, which represent a novel approach to SCD treatment [210]. Additionally, researchers are exploring combination therapies to address the complex nature of SCD manifestations [213,214]. While gene therapy holds promise for a cure, its widespread application remains limited due to technical and cost challenges [215]. These developments offer hope for improved quality of life and survival for SCD patients.

**Figure 3:**
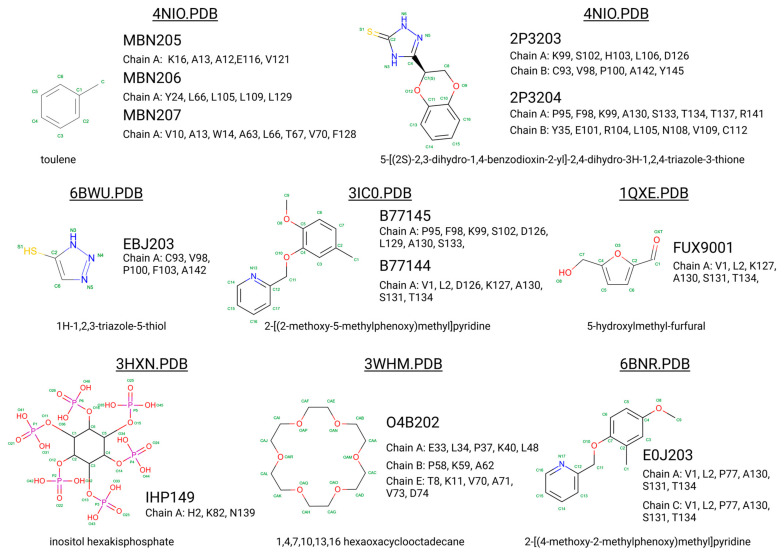
Examples of various small molecules binding to hemoglobin and interacting with specific hemoglobin residues. The figure includes the chemical structures of the ligands, such as toluene, 1H-1,2,3-triazole-5-thiol, inositol hexakisphosphate, and others. Additionally, it lists the specific residues in one of the globin chains that interact with these small molecules. The corresponding PDB codes for the hemoglobin-small molecule complexes are also provided for reference.

Hemoglobin exhibits remarkable versatility in binding a wide variety of small molecules, reflecting its functional and structural adaptability (Table 2, Figure 3). Figure 3 showcases examples of these interactions, supported by three-dimensional structures available in the Protein Data Bank (PDB). Representative hemoglobin-small molecule complexes include toluene and 2,3-dihydro-1,4-benzodioxin-2-yl)-4H-1,2,4-triazol-3-yl)disulfide (TD1, PDB: 4NI0), 1H-1,2,3-triazole-5-thiol (TD3, PDB: 6BWU), 2-[(2-methoxy-5-methylphenoxy)methyl]pyridine (INN-298, PDB: 3IC0), 5-hydroxymethyl-furfural (5HMF, PDB: 1QXE), inositol hexakisphosphate (IHP, PDB: 3HXN), 1,4,7,10,13,16-hexaoxacyclooctadecane (18-crown-6, PDB: 3WHM), 2-[(4-methoxy-2-methylphenoxy)methyl]pyridine (vanillin derivative screening, INN-310, PDB: 6BNR), 2-hydroxy-6-((6-(hydroxymethyl)pyridin-2-yl)methoxy)benzaldehyde (VZHE-039, PDB: 6XD6), 2-[4-({[(3,5-dichlorophenyl)amino]carbonyl}amino)phenoxy]-2-methylpropanoic acid (L35, PDB: 2D5Z), (2R,4S)-2-hydroxy-1-[2-hydroxy-3-(trifluoromethyl)phenyl]-4-[4-(trifluoromethyl)phenyl]pyrrolidine-3,5-dione (GBT440, Voxeltor, PDB: 5E83) and 6-{(1S)-1-[(2-amino-6-fluoroquinolin-3-yl)oxy]ethyl}-5-(1H-pyrazol-1-yl)pyridin-2(1H)-one (compound 23, PF-07059013, PDB: 7JY3). While these examples illustrate the chemical diversity of molecules that bind to hemoglobin, they represent only a subset of the available structural data. Not all hemoglobin–ligand structures have been published, and the presented examples are chosen to demonstrate the range of chemical scaffolds and binding interactions observed. Importantly, these small molecules interact with specific residues in hemoglobin chains, underscoring the molecular specificity of binding. Some molecules, such as TD1 (PDB: 4NI0), interact with residues in both α and β chains of hemoglobin, while others bind selectively to residues within a single chain. This variability highlights the dynamic nature of hemoglobin’s interaction with ligands, as well as its potential for modulating functional states through small-molecule binding. The provided examples demonstrate hemoglobin’s capacity to bind chemically distinct compounds, offering valuable insights for designing therapeutics targeting hemoglobin-related disorders or for understanding its physiological roles in molecular detail.

Compounds like TD7, VZHE039, and their nitric oxide-releasing derivatives have shown promising antisickling properties by increasing hemoglobin’s oxygen affinity and disrupting HbS polymerization [216]. TD-1, a small molecule identified through screening, demonstrated dual allosteric and antioxidant effects by binding to βCys93 and βCys112, stabilizing hemoglobin’s relaxed state and reducing oxidative changes [217,218]. Other compounds like TD-3 and hydroxyurea also target βCys93, providing both antioxidant and antisickling benefits [219]. These studies have elucidated the mechanisms of action for various antisickling agents, including their effects on oxygen affinity, HbS polymerization, and oxidative stress reduction. Further research on antisickling agents targeting hemoglobin’s β-Cys93 has shown promising results for treating sickle cell disease. Other thiol reagents reacting with β-Cys93 have shown varying antisickling effects [220,221]. Overall, increasing hemoglobin’s oxygen affinity represents a promising therapeutic approach for sickle cell disease.

5-hydroxymethyl-2-furfural (5HMF) is a promising compound for treating sickle cell disease. It forms a Schiff-base adduct with hemoglobin, increasing oxygen affinity and inhibiting red blood cell sickling [222,223]. 5HMF demonstrates dose-dependent effects on hemoglobin oxygen affinity in phase 1 clinical trials [224]. It improves cardiac function during severe hypoxia [225] and reduces deoxygenation-induced dehydration of red blood cells by inhibiting cation conductance and Ca^2+^-activated K^+^ channels [226]. Researchers have developed ester and ether derivatives of 5HMF with enhanced antisickling properties [223] and explored nitric oxide-releasing prodrugs [227]. The mechanism of action involves stabilizing the R state of hemoglobin, which has higher oxygen affinity and slower polymerization kinetics [228,229]. These findings support 5HMF as a potential therapeutic agent for sickle cell disease.

Hb plays a crucial role in nitric oxide metabolism and oxygen transport. Hb can form various NO-related compounds, including nitrosylated Hb (HbNO) and S-nitrosohemoglobin (SNO-Hb) [230,231]. These compounds are involved in regulating vascular function and oxygen delivery [15,232]. The interaction between Hb and NO is complex, involving redox reactions and allosteric effects [230,233]. SNO-Hb has been proposed as a key mediator of NO transport and bioactivity, with its formation and release coupled with oxygen saturation [15,234]. However, the precise mechanisms of NO activity preservation and regulation by Hb remain debated [235,236]. Understanding these interactions is crucial for developing transfusion therapeutics and treating various cardiovascular diseases [232].

Recent research on SCD has also focused on developing aromatic aldehydes as potential treatments by increasing hemoglobin oxygen affinity and inhibiting red blood cell sickling. Novel compounds, including pyridyl derivatives of vanillin and azolylacryloyl derivatives, have shown improved potency, metabolic stability, and sustained antisickling effects in vitro [237,238]. Some compounds exhibit both oxygen-dependent and oxygen-independent antisickling mechanisms, potentially disrupting key intermolecular contacts necessary for hemoglobin S polymer formation. Crystal structure studies have provided insights into the binding modes and mechanisms of action of these compounds [238]. The development of hemoglobin modulators represents a promising approach to treating SCD, with ongoing research and growing intellectual property in this field [239]. These advancements complement other therapeutic strategies, including targeting cellular adhesion, inflammation, and oxidant injury.

PF-07059013 (compound **23**) is a noncovalent hemoglobin modulator that has advanced to phase 1 clinical trials for sickle cell disease (SCD) treatment. It demonstrated a 37.8% reduction in red blood cell sickling in a mouse model [240]. This compound represents a new approach to SCD treatment, targeting the root cause of the disease: hemoglobin S polymerization [241]. Unlike earlier covalent modifiers with potential safety concerns, PF-07059013 binds noncovalently to hemoglobin [242]. Similar approaches, such as GBT440, have shown promise in preclinical testing [243,244]. These developments are part of a broader effort to create new SCD therapies, including fetal hemoglobin inducers and agents targeting cellular adhesion, inflammation, and vascular tone [245]. Such advancements offer hope for improved SCD management beyond current treatments like hydroxyurea and blood transfusions [246].

Vanillin derivatives have shown promise as potential treatments for sickle cell disease and other conditions. Novel compounds like SAJ-009, SAJ-310, and SAJ-270 demonstrated improved binding and pharmacokinetic properties compared to vanillin, with enhanced allosteric and antisickling effects [238]. SAJ-310 exhibited sustained antisickling activity and potential metabolic stability [247]. Vanillin derivatives also showed antioxidant properties and inhibitory activity against acetylcholinesterase and β-amyloid aggregation, suggesting potential applications in Alzheimer’s disease treatment [248]. Additionally, vanillin demonstrated analgesic effects on mechanical allodynia in a neuropathic pain model [249] and inhibitory activity against mushroom tyrosinase [250]. The compound’s ability to react covalently with sickle hemoglobin and inhibit cell sickling supports its potential as a treatment for sickle cell anemia [251]. Vanillin’s diverse pharmacological activities and safety profile make it a promising candidate for further research and development.

VZHE-039 is a novel antisickling agent that exhibits both oxygen-dependent and oxygen-independent mechanisms of action [252]. In this study, VZHE-039 demonstrates enhanced pharmacologic activities and metabolic stability. Its oxygen-independent activity is attributed to interactions with the αF-helix of hemoglobin, potentially destabilizing the sickle hemoglobin polymer [253]. Other antisickling agents, such as voxelotor and FT-4202, also show promise in treating sickle cell disease by increasing hemoglobin oxygen affinity.

Crown ethers, cyclic polyethers with oxygen atoms, have shown potential in modulating protein surface properties and interactions [254]. They can bind to metal ions and positively charged amino acid side chains, affecting protein behavior such as oligomerization and crystallization. In hemoglobin research, crown ethers and related compounds have been explored for their ability to modify hemoglobin’s oxygen-binding properties. Acylation of hemoglobin with polyoxyethylene derivatives has been shown to alter its oxygen affinity [255]. Other synthetic allosteric effectors, such as RSR-4 and RSR-13, have demonstrated the ability to shift hemoglobin’s allosteric equilibrium towards the low-affinity T-state [256]. These studies highlight the potential of crown ethers and related compounds in modulating hemoglobin function and developing novel therapeutic approaches.

Complex interactions between hemoglobin (Hb) can occur also with natural molecules such as sphingosine 1-phosphate (S1P) and lipopolysaccharide (LPS). S1P is a bioactive lipid involved in immune responses, cell growth, and survival. It has been found to bind specifically to deoxygenated sickle hemoglobin (HbS), the form of hemoglobin that promotes the sickling of red blood cells in sickle cell disease. When S1P binds to HbS, it alters glucose metabolism and increases oxidative stress, both of which are significant contributors to the pathology of sickle cell disease. Altered glucose metabolism disrupts energy production in red blood cells, while increased oxidative stress leads to cellular damage and inflammation, further exacerbating the disease’s symptoms [257]. Hb also interacts with lipopolysaccharide (LPS), a component of the outer membrane of Gram-negative bacteria, enhancing its biological activity and potentially contributing to the immune defense against bacterial infections. This interaction suggests that hemoglobin may play a role beyond oxygen transport, acting as a modulator of the immune response [258,259]. Multiple binding sites for LPS have been identified on both the α and β-subunits of hemoglobin, highlighting the molecule’s multifunctional nature and its potential importance in pathogen defense [259,260]. Hemoglobin also binds to erythrocyte membranes, an interaction crucial for the stability and integrity of red blood cells. This binding is driven by electrostatic forces influenced by factors such as pH and ionic strength, which may vary under different physiological conditions, such as during oxygenation/deoxygenation cycles [261,262]. A critical protein involved in this interaction is band 3 protein, a major structural component of the erythrocyte membrane. The acidic N-terminal segment of the cytoplasmic domain of band 3 serves as a primary binding site for hemoglobin [167]. This interaction is essential for maintaining the structural integrity of red blood cells and regulating their lifespan. Disruptions in this binding can lead to membrane instability and hemolysis, a hallmark of hemolytic anemias such as sickle cell disease. In pathological conditions like sickle cell disease, these hemoglobin interactions are further altered, contributing to disease progression. The S1P-Hb interaction exacerbates oxidative stress, worsening the symptoms of sickle cell disease. The LPS-Hb interaction could play a dual role in immune defense and the potential triggering of exaggerated immune responses.

Finally, hemoglobin’s binding to the erythrocyte membrane via band 3 protein is critical for cell stability, and disruptions in this interaction could contribute to the shortened lifespan of red blood cells seen in sickle cell disease and other hemoglobinopathies. In summary, these complex interactions between hemoglobin, S1P, LPS, and the erythrocyte membrane are critical for both normal physiological processes and pathological conditions, especially sickle cell disease.

Voxelotor is a novel oral treatment for SCD that binds to hemoglobin, increasing its oxygen affinity and preventing the polymerization of sickle hemoglobin (HbS) [263,264]. By forming a reversible covalent bond with hemoglobin, voxelotor reduces the sickling of red blood cells and interrupts the molecular pathogenesis of SCD. Clinical trials have demonstrated that voxelotor increases hemoglobin concentration, reduces hemolysis, and improves hematological parameters in SCD patients [265,266]. The drug has shown a dose-dependent pharmacokinetic and pharmacodynamic response and is well-tolerated [267]. Voxelotor represents a new disease-modifying approach to SCD treatment, targeting the root cause of the disease [268].

## 3. Search for Potentially New Hb Stabilisers: Hemoglobin Binding Proteins

Innovative approaches to stabilizing hemoglobin leverage specialized molecular chaperones, engineered enzymes, and highly specific binding agents to counter the structural vulnerabilities of various hemoglobin variants. These methods go beyond traditional therapies by focusing directly on the molecular factors that lead to hemoglobin instability, aggregation, or ineffective oxygen transport. For example, alpha-hemoglobin-stabilizing protein (AHSP) is an erythroid-specific chaperone that binds free α-globin chains and prevents their precipitation [168,170,269,270,271,272]. In conditions where the balance of α- and β-globin is disrupted (e.g., α-thalassemia or other unstable hemoglobin variants), AHSP can mitigate the harmful accumulation of unpaired α-globin, which would otherwise form inclusion bodies, generate oxidative stress, and shorten red blood cell lifespan. By enhancing AHSP expression or designing small molecules that mimic its stabilizing interface, one could restore proper globin chain stoichiometry and improve red blood cell survival. For instance, future therapies might involve drugs that upregulate AHSP in erythroid progenitors or engineered AHSP analogs that bind mutated α-globins more efficiently, thus preventing hemoglobin instability at its source.

Enzymes like falcilysin, a metalloprotease from the malaria parasite Plasmodium falciparum, also inspire innovative strategies. Although falcilysin’s natural function is to degrade hemoglobin within the parasite’s food vacuole, its specificity and mechanism highlight how selective proteolysis could be harnessed or inhibited to influence hemoglobin stability [273,274,275,276,277]. Inhibiting falcilysin is already a potential antimalarial strategy, but repurposing or engineering similar proteolytic activities could help clear unstable hemoglobin fragments in other contexts. For example, engineered human proteases or inhibitors could be designed to selectively remove or neutralize harmful, partially denatured hemoglobin species in patients with unstable variants. This would prevent the formation of damaging aggregates, such as Heinz bodies, and reduce the oxidative burden on red blood cells.

Nanobodies, small antibody-derived fragments with high specificity and affinity, represent another frontier. By targeting key regulators of hemoglobin gene expression, such as BCL11A, nanobodies can potentially shift the balance toward fetal hemoglobin (HbF) production, alleviating symptoms in disorders like sickle cell disease and β-thalassemia [278,279]. Beyond gene regulation, nanobodies like NbE11 that precisely bind human hemoglobin have immediate applications in diagnostics. Their exceptional specificity could be adapted for therapeutic delivery systems that “escort” stabilizing compounds directly to unstable hemoglobin variants within red blood cells, thus minimizing off-target effects. For instance, a nanobody could be fused to a small molecule or peptide stabilizer, ensuring the agent reaches and binds specifically to the defective hemoglobin subunits, improving stability and function without altering healthy hemoglobin.

In addition, molecularly imprinted polymeric nanoparticles provide a synthetic approach to selectively bind and purify human hemoglobin [280]. These “plastic antibodies” could be tailored to recognize and capture unstable hemoglobin variants from patient samples, aiding in precise diagnostics and screening assays. By modifying the imprinting process, one might even create nanoparticles that deliver stabilizing agents to red blood cells or sequester oxidized hemoglobin species, improving red cell viability.

Collectively, these strategies—enhancing AHSP function, leveraging proteases like falcilysin for targeted intervention, employing nanobody-based precision tools, and using molecular imprinting technologies—offer a sophisticated toolkit for addressing hemoglobin instability at a molecular level. By focusing on the structural and mechanistic aspects of hemoglobin, these innovative approaches have the potential to yield more effective, variant-specific treatments and diagnostic platforms, ultimately improving patient outcomes in hemoglobinopathies.

Innovative approaches targeting hemoglobin stability and function may enhance protein platforms for protein engineering by the use of protein chaperones, enzymes like falcilysin, and nanobodies.

## 4. Conclusions

Hemoglobinopathies such as sickle cell disease and thalassemias are driven by diverse hemoglobin variants, posing challenges for universal therapies. This review highlights promising approaches like small-molecule binders (e.g., voxelotor) and peptides that enhance hemoglobin stability, increase oxygen affinity, and reduce oxidative stress [210,219,263,264,265]. Combining these therapies with antioxidants offers additional potential [181,182,183,184]. Future research should focus on discovering new hemoglobin-targeting molecules, utilizing advanced techniques like time-resolved X-ray diffraction [281] and in situ crystallization [282], and refining gene-editing tools such as CRISPR/Cas9 for curative treatments [118,119]. By integrating these strategies, tailored and effective therapies can significantly improve outcomes for hemoglobinopathy patients.

## Figures and Tables

**Table 2:** Overview of small molecules and binders targeting hemoglobin: interactions and therapeutic or research potential. The exact appearance times of these compounds may vary, as they depend on how “appearance” is defined—whether it refers to their initial discovery, release date, or first mention in research publications. For this overview, we assume the timeline is based on the release date and/or first mention in relevant research papers.

Small Molecule/Binder	Appearance Time	Main Interaction with Hemoglobin	Therapeutic or Research Impact
VZHE-039 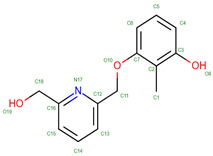	2020	Enhances oxygen transport efficiency	Potential therapeutic agent to improve oxygen delivery
Compound **23** (PF-07059013) 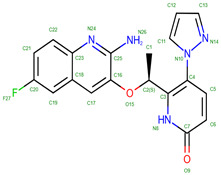	2020	Noncovalent binder that enhances hemoglobin stability	Explored for reducing sickling in sickle cell disease
INN-310 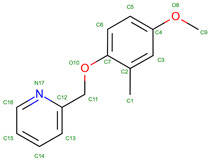	2017	Vanillin derivative that influences hemoglobin stability	Explored for impacts on oxygen affinity and hemoglobin stability
TD3 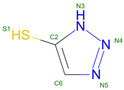	2017	Affects oxygen binding dynamics	Potential for therapeutic use in modifying hemoglobin function
GBT440 (Voxeltor) 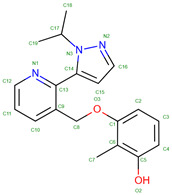	2015	Increases oxygen affinity to prevent sickle hemoglobin polymerization	Used in treating sickle cell disease
18-crown-6 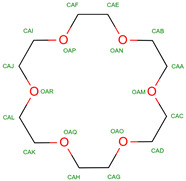	2013	Alters oxygen-binding properties	Used in studies for potential modulation of hemoglobin function
Toluene 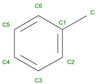	2013	Solvent in structural studies	Used to understand hemoglobin structure
TD1 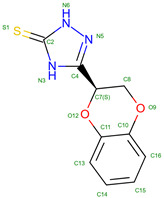	2013	Alters hemoglobin’s structural stability	Studied for potential therapeutic impacts on hemoglobin function
INN-298 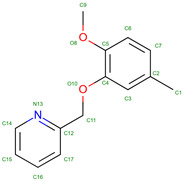	2005	Modifies hemoglobin function	Investigated for its effects on hemoglobin and potential treatments
L35 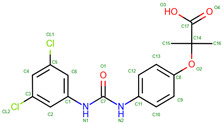	2005	Modifies oxygen affinity and function	Investigated for its potential to treat hemoglobinopathies
5-Hydroxymethylfurfural (5HMF) 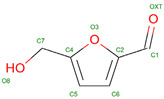	2003	Increases oxygen affinity, reducing sickling	Explored for treatment of sickle cell disease
Inositol Hexakisphosphate (IHP) 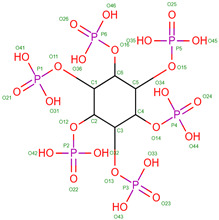	1974	Stabilizes deoxyhemoglobin, reduces oxygen affinity	Research tool for studying oxygen release mechanics

## Abbreviations

The following abbreviations are used in this manuscript:

Hb	Hemoglobin
SNO	S-nitrosohemoglobin
BPG	Bisphosphoglycerate
HbF	Fetal Hemoglobin
HbA	Adult Hemoglobin
HbC	Hemoglobin C
HbD	Hemoglobin D
HbE	Hemoglobin E
Hb CS	Hb Constant Spring
ACMG	American College of Medical Genetics and Genomics
SCD	Sickle Cell Disease
RBC	Red Blood Cell
ROS	Reactive Oxygen Species
AHSP	α-Hemoglobin Stabilizing Protein
G6PD	Glucose-6-phosphate Dehydrogenase
NAC	N-acetylcysteine
ALAS-1	Aminolevulinic Acid Synthase
S1P	Sphingosine 1-phosphate
LPS	Lipopolysaccharide
